# Edible oils as a co-extractant for the supercritical carbon dioxide extraction of flavonoids from propolis

**DOI:** 10.1371/journal.pone.0266673

**Published:** 2022-04-12

**Authors:** Parveen Devi Pattiram, Faridah Abas, Norhidayah Suleiman, Ezzat Mohamad Azman, Gun Hean Chong

**Affiliations:** 1 Faculty of Food Science and Technology, Universiti Putra Malaysia, UPM Serdang, Selangor, Malaysia; 2 Supercritical Fluid Center, Faculty of Food Science and Technology, Universiti Putra Malaysia, UPM Serdang, Selangor, Malaysia; Institute for Biological Research, University of Belgrade, SERBIA

## Abstract

Propolis is a good source for flavonoids, however, their recovery is challenging, as it is a waxy material. This study investigated edible oils virgin coconut oil (VCO), corn oil (CO), and ghee (G) as co-extractants for the supercritical carbon dioxide (scCO_2_) extraction of flavonoids from the propolis. The extraction of flavonoids using 20% VCO as co-extractant with scCO_2_ (25 g/min) for 210 min at 150 bar and 50°C was found to be the most appropriate, yielding a total flavonoid content (TFC) of 11.7 mg/g and 25% TFC recovery. At a higher temperature (60°C) and pressure (250 bar and 350 bar), the propolis became softer and compressed causing the extractions to retrograde. The extraction curves correlated to the diffusion model with 1.6% (AARD). The matrix diffusivities increased from 4.7 × 10^−11^ m^2^/s (scCO_2_) to 6.9 × 10^−11^–21.4 × 10^−11^ m^2^/s upon the addition of edible oils. Thus, edible oils could be used with scCO_2_ to improve the flavonoid extraction from propolis.

## Introduction

Bees use propolis in their hives as protection against predators and microorganisms, repair damage, and as a thermal isolator [1]. It is a natural resinous material that honeybees collect from various plants and mix with beeswax and salivary enzymes [2]. Propolis extracts have been shown to possess antioxidant, anti-inflammatory, antibacterial, anticarcinogenic, and antihepatotoxic activities [2]. Polyphenol compounds, namely phenolic acids, and flavonoids, found in the propolis contribute to these therapeutic activities [3].

The extraction of bioactive compounds from propolis is usually performed with ethanol [4] and mixture of ethanol and water (70:30 v/v) [5]. Some studies have applied external forces such as ultrasonic [6] and microwave [7] to improve extraction rate. However, these methods need a downstream process for removing solvent and residual of solvent in extract has always been concerned. Supercritical carbon dioxide (scCO_2_) is explored for extracting the bioactive compounds from propolis [8], but pure scCO_2_ is not suitable because of its chemical characteristics and the physical properties of the propolis [9]. As the propolis consists of 30% wax [10], organic solvents are needed to swell the matrix, increase the contact surface area, and dissolve the solutes [11]. Machado *et al*. reported that with 1% of ethanol (co-solvent) in scCO_2_ has improved the extraction of hydrophilic compounds from propolis [12]. Additions up to 7% of ethanol also improve the extraction yield from hard and sticky propolis [13].

Vegetable oils have been used as a solvent in extraction and purification processes [14] and are particularly successful for long extraction times [15]. One of the advantages of this approach is that a subsequent process for the separation of these oils from the extracts is not required [7]. By using edible oil as solvent, the study conducted by Tosi *et al*. has shown that the extract of propolis has a wide range of antimicrobial activity [16]. Carvalho *et al*. has also reported that the inhibition Sarcoma 180 tumour growth in mice is the same for ethanol extract and canola oil extract of propolis [17]. Some researchers have used scCO_2_ to intensify the extraction when vegetable oil was used as the solvent. For example, Krichnavaruk *et al*. used soybean oil and olive oil with scCO_2_ to extract *astaxanthin* from *Haematococcus pluvialis*, reporting 36% and 51% extraction efficiency for soybean oil and olive oil, respectively [18]. The extraction efficiency of lycopene from tomato peel is 60% for hazelnut oil [19] and 56% for tomato seed oil [20] in combination with scCO_2_. For marigold, carotenoid extraction was increased two-fold by the addition of canola oil to scCO_2,_ and more than a four-fold higher lutein yield was obtained than with scCO_2_ alone [21]. The maximum yield of 10.3 mg lutein/g marigold was achieved using soybean oil as a co-solvent [22] and that of lutein esters (10.2 mg/g of marigold) with the addition of medium-chain triglycerides [23].

One of the challenges in propolis extraction is the high wax content that retards the extraction process; therefore, this study investigated the role of edible oils namely ghee (G), virgin coconut oil (VCO), and corn oil (CO) in assisting scCO_2_ for the extraction of flavonoids from propolis. The diffusion model was used to correlate the overall extraction curves (OEC) and estimated the diffusivity of the solutes into the solvent in the presence of edible oil. The extracts were also analysed for the tentative flavonoids.

## Materials and methods

### Materials

Virgin coconut oil from Orifera^TM^ (Malaysia), QBB pure ghee (Malaysia), and Mazola^®^ corn oil (Malaysia) were used in this study. Liquid CO_2_ (purity of 99.999%) was purchased from Mox-Linde Gases Sdn. Bhd. (Malaysia). Quercetin, Folin-Ciocalteau reagent, aluminium chloride reagent, n-hexane (HPLC grade), acetonitrile (HPLC grade), formic acid (0.1%), hydrochloric acid (37%), sulfuric acid (98%), and methanol (HPLC grade) were purchased from Scienfield Sdn. Bhd. Malaysia. Purified water from the Milli Q system was used in all the experiments. The brown honey bees (*Apis mellifera*) propolis species sample was obtained from Honey Bee Farm, Selangor, Malaysia was vacuum-packed in an aluminum pouch. It was stored at ca. −20°C for further analysis.

### Sample preparation

Propolis was removed from the freezer and ground immediately with a marble mortar and pestle at room temperature (ca. 20°C), then sieved to obtain particles sized 700 μm to 400 μm with a sieve shaker (AS 200, Retsch, UK); the nominal size was 500 μm.

### Moisture content analysis

The moisture content of propolis was determined with a moisture analyser (Kern, DBS 60–3, UK) in triplicate at 105°C for approximately 10 min. The moisture content percentage was calculated based on the sample final and initial mass [24].

### Ash content analysis

The ash content was determined according to the AOAC method [25] with some modifications. First, 10 g of grounded raw propolis was placed in a crucible and heated in a muffle furnace (Thermolyne™, Maxi-Mix III, USA) at ca. 550°C for 24 h to ensure that it turned grey. The final weight of the ash with the crucible and lid was recorded (n = 3) and the ash content was calculated as follows:

$$Ash\;(\%)=\frac{mass\;of\;ash}{mass\;of\;sample}\;\times 100\%$$ (1)

### Crude fat content analysis

This analysis was performed following the standard method [25]. First, 10 g of grounded raw propolis was placed in a thimble, and then the thimble was inserted into the Soxhlet apparatus in a water bath (RE300, Stuart, UK) at ca. 40°C, before the addition of 400 mL. The procedure was conducted for 8 h and the n-hexane was evaporated from the round-bottomed flask using a rotary evaporator (RE300, Stuart, UK) at ca. 60°C. The flask was placed in an oven (UFP600, Memmert, Germany) at ca. 105°C for 5 min for further evaporation, and then weighed until a constant mass was achieved. The experiment was conducted in triplicate. The percentage of fat content was calculated as follows:

$$Oil\;content\;(\%)=\frac{mass\;of\;oil\;collected}{mass\;of\;sample}\;\times 100\%$$ (2)

### Crude protein analysis

The crude protein content was determined according to AOAC 2000 [25] with minor modifications. Briefly, 10 g of grounded raw propolis was placed in a micro Kjeldahl test tube and 2.5 mL of concentrated sulfuric acid was added. The solution was boiled until it became clear and exhibited a blue/green colour. Next, 10 mL of distilled water was added, followed by the addition of a 45% NaOH solution to separate the solution into two layers, followed by reverse titration with hydrochloric acid (0.5 N). The percentage of protein in the sample was obtained by multiplying the conversion factor (6.38) by the nitrogen percentage. The analysis was conducted in triplicate.

### Crude fiber analysis

First, 10 g of grounded raw propolis was hydrolysed with aqueous sulfuric acid (200 mL, 0.25 N), then filtered through filter paper Whatman No. 541. The residue was rinsed with boiled water to remove the acid and dried in an oven (UFP600, Memmert, Germany) for 7 h at ca. 105°C. The mass of the crude fiber was the difference between the dried sample residue and the ash [25].

### Wax content analysis

The wax content of raw propolis was determined using the density method [26]. First, 20 mL of deionized water was added to 10 g of ground raw propolis. The sample was heated using a water bath (RE300, Stuart, UK) at ca. 100°C for 5 min, then cooled to room temperature (ca. 25°C). A three-layered solution was obtained, with beeswax in the top layer, followed by a water layer, and the bottom layer was de-waxed propolis. The upper layer was transferred to filter paper (Whatman filter paper No. 41) for the removal of access water. The analysis was performed in triplicate. The percentage of wax was calculated as follows:

$$Wax\;(\%)=\frac{mass\;of\;wax}{mass\;of\;sample}\;\times 100\%$$ (3)

### Extraction of flavonoids with supercritical carbon dioxide (scCO_2_)

**Fig 1:**
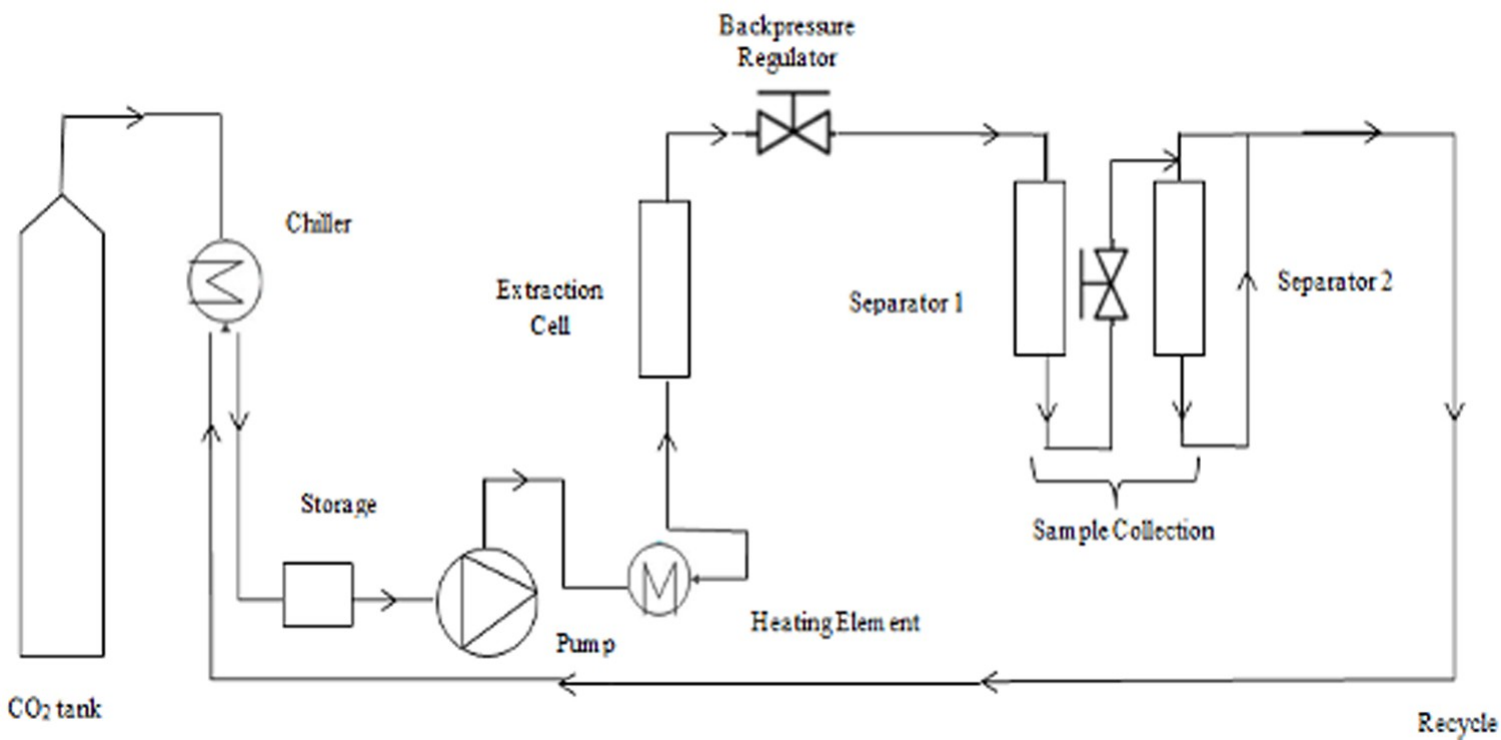
Schematic representation of the supercritical carbon dioxide extraction unit.

A schematic representation of the scCO_2_ extractor is shown in Fig 1 and the detailed scCO_2_ extraction methodology was explained in the previous study [27]. Briefly, 20% mass ratio of edible oil was dripped and mixed with the ground propolis (20 g) and edible oil was adsorbed. The oil ratio was fixed at 20% because the oil was not adsorbed by propolis when more oil was added. The mixture was filled into a nylon bag (15 μm mesh) and loaded into the extraction vessel (1 L, diameter = 0.08 m, height = 0.21 m). The extract was collected from separator 1 and 2, and most of extract was collected from separator 1. The mass of extract (separator 1 and separator 2) was recorded over time until the extraction was completed. The extractions conditions were tabulated in Table 2. 150 bar, 50°C and CO_2_ flowrate 25 g/min were used as the basic extraction conditions because this was the minimal conditions that flowable extract could be obtained. The experiments were conducted in duplicate. The edible oils used in this study were ghee (G), virgin coconut oil (VCO), and corn oil (CO), which are short-, medium-, and long-chain triglycerides, respectively. The same methodology was applied when methanol was used as co-solvent. Extraction yields were calculated as follows:

$$\mathrm{Yield}(\%)=\frac{\mathrm{mass}\;\mathrm{of}\;\mathrm{extract}}{\mathrm{mass}\;\mathrm{of}\;\mathrm{feed}}\times 100\%$$ (4)

### Extraction of flavonoids with edible oil

4 g of G, VCO and CO oil were dripped and mixed separately with 20 g of propolis in a centrifuge tube, and soaked in water bath (RE300, Stuart, UK) for 2.5 h at ca. 50°C. The mixture was centrifuged (Agilent Technologies Inc., USA) at the speed of 20 × 10 g for 60 min [28] and the supernatant was decanted before the extracts were stored at ca. -20°C for further analyses. The experiments were conducted in duplicate. The mass ratio of oil and propolis was maintained as performed in scCO_2_ extraction.

### Extraction of flavonoids with methanol

First, 20 g of ground propolis was extracted with 25 mL of methanol for 2.5 h at ca. 50°C with a magnetic stirrer (Thermolyne™, Maxi-Mix III, USA). Subsequently, the extract was filtered using Whatman filter paper No. 41 and assisted with a vacuum pump (RE300, Stuart, UK) at 30 kPa. After filtration, methanol was removed with a vacuum rotary evaporator at 35 kPa (Model RE401, Stuart, Tokyo) at ca. 50°C. The extract was left in an oven (Memmert GmbH, Germany) for 24 h at ca. 30°C to ensure that methanol was removed completely [29]. The experiments were conducted in duplicate. The propolis extracts were stored at ca. -20°C for further analyses.

### Determination of total flavonoids

The total flavonoid content (TFC) of the extract was determined using the aluminium chloride method [30]. First, 0.1 mL of the extract was mixed with 1 mL of AlCl_3_ (2% w/v, in methanol). After 40 min at room temperature (ca. 25°C), the absorbance was read with a spectrophotometer (Thermo scientific, Genesys, UK) at 415 nm. Quercetin was used as the standard to produce the calibration curve. The TFC was expressed as the mass of Quercetin Equivalent in the dry extract (mg/g). The experiments were conducted in triplicate, and the data were interpreted further as follows:

$$\mathrm{TFC}\;\mathrm{recovery}\;(\%)=\frac{\mathrm{concentration}\;\mathrm{of}\;\mathrm{TFC}\;\mathrm{in}\;\mathrm{the}\;\mathrm{extract}}{\mathrm{concentration}\;\mathrm{of}\;\mathrm{TFC}\;\mathrm{in}\;\mathrm{the}\;\mathrm{control}}\times 100\%$$ (5)

$$\mathrm{Extraction}\;\mathrm{effectiveness}\;\left(\frac{g}{g}\right)=\frac{\mathrm{mass}\;\mathrm{of}\;\mathrm{TFC}\;\mathrm{in}\;\mathrm{the}\;\mathrm{extract}}{\mathrm{mass}\;\mathrm{of}\;\mathrm{solvent}\;\mathrm{used}}\;$$ (6)

$$Co-extractant\;effectiveness\;\left(\frac{g}{g}\right)=\frac{mass\;of\;TFC\;in\;the\;extract}{mass\;of\;co-extractant\;used}$$ (7)

where *control* is the methanol–propolis extract and *co-extractant* is the edible oil used with supercritical carbon dioxide.

### Identification of tentative flavonoid compounds

The analyses were performed using an Agilent 1290 RRLC (Rapid Resolution Liquid Chromatography) series equipped with a 6550 IFunnel LC/MSQ-ToF System (Agilent, USA). The samples were filtered through a 0.2-μm membrane filter. The mobile phase comprised (A) 100% water and (B) formic acid (0.1%, 1 mL) in acetonitrile (100%, 1000 mL). The solvent gradient started with 5% B (0.5 min), 5%–30% B (45 min), 30%–60% B (4 min), 60%–90% B (5 min), 95–5% B (1 min), and 5% B for the calibration (5 min). The mass spectrometry parameter was in the positive ion mode. The gas temperature was 290°C with the flow of 11 L/min, nebulizer pressure (N_2_) was 35 psig, sheath gas at 11 L/min at 300°C. The capillary voltage was set at 3000 V, nozzle voltage at 2000 V, and fragmentor voltage at 175 V. The column was an Agilent Zorbox Rapid Resolution High Definition Eclipse Plus C18 (2.1 mm × 150 mm × 1.8 μm). The total run time was 20 min, with a 3-μL injection volume [31]. The tentatively compounds were determined using the internal library, Knapsack core system library and National Institute of Standard and Technology (NIST). This was done in accordance with the parameters of selectivity, linearity, precision, accuracy, detection limits and quantification limits.

### Overall extraction curve modelling

The overall extraction curve (OEC) was prepared by plotting the extraction yield over time. The front part of the curve, which was assumed to achieve equilibrium, was used to estimate the solubility of the extract in scCO_2_ by using Eq (8).


$$Solubility\;\left(\frac{g}{g}\right)=\frac{mass\;of\;extract}{mass\;of\;carbon\;dioxide\;used}$$ (8)

The OECs were also correlated with the diffusion model to investigate the edible oils’ effects on the solute diffusion in the matrix [32]. The corresponding equation is as follows:

$$\frac{m_{t}}{m_{o}}\;=1-\frac{6}{\pi }\;\sum_{n=1}^{\infty }\frac{1}{n^{2}}\exp(\frac{-n^{2}\pi^{2}D_{m}t}{r^{2}})$$ (9)

where *m*_*t*_ is the mass of the extract at time *t*, *m*_*o*_ is the extractable solute, *D*_*m*_ is the matrix diffusivity, and the particles are assumed to be spherical. The model was solved with the Microsoft Excel (Microsoft, USA) non-linear solver, and the objective function used was as follows:

$$AARD\;=\sum_{i\;=1}^{n}\frac{{Value}_{experimental}-{Value}_{calculated}}{{Value}_{experimental}}\times 100\%\;$$ (10)

### Statistical analysis

The results were analysed separately using Analysis of variance (ANOVA) at a 5% uncertainty level in Minitab 17.1 statistical software (Minitab, USA). The Tukey test was applied to determine the significant difference between the extraction methods.

## Results and discussion

### Proximate analyses of the raw propolis

Table 1 shows the composition of the propolis used in this study, with the major components being fiber (31.3%), wax (31.8%), and fat (19.2%). The wax content was similar to that reported in the review by Syed *et al*. [33], which considered a propolis sample with 30% bee wax as the major components. The compositions obtained in this study were similar from those found in the literature from other samples of propolis, which presented of total ash content (1.6%–4.4%), (6.5%–32.3%) for crude fat [12]. Based on Attia *et al*. the variations found due to the type of propolis studied, geographical region, environmental conditions, and the collection period of the sample [34].

**Table 1:** Composition of propolis used in this study.

Proximate compositions	Composition (%)		
	This study		Reference
Moisture	9.7 ± 0.06	7.1 [12]	
Ash	6.2 ± 0.04	3.1 [12]	
Crude fat	18.2 ± 0.02	45.6 [12]	
Crude protein	2.9 ± 0.07	10.0 [12]	
Crude fiber	31.3 ± 0.04	20.9 [12]	
Wax	31.8 ± 0.03	30 [32]	

### Extraction with edible oils

The extraction of TFC with edible oils is presented in Table 2 (Run 1, Run 2, and Run 3). The extract obtained from VCO was the highest in TFC, followed by CO and G. The TFC recoveries were only up to 1%. These results showed that the 20% edible oil to propolis mass ratio at 50°C barely managed to extract TFC from the propolis. However, the concentration of TFC could be increased with prolong the extraction time. As reported by Pujirahayu *et al*. that with the maceration of propolis with VCO for 7 days able to obtain 2.5 mg/g of TFC in the extract [35]. A patent [36] also has reported that vegetable oils can be used in extracting artepillin C from propolis. Thus, we inferred that VCO might be able to extract flavonoids from propolis but the limitations could be attributed to the properties of propolis and the mass of VCO used in this study.

**Table 2:** Comparison of methods for the extraction of flavonoids from the propolis.

Run	Pressure (bar)	Temperature (°C)	Solvent	Yield (%)	Solubility (g/g)×10^−4^	TFC in extract (mg/g)	TFC recovery (%)	TFC extraction effectiveness ×10^−7^(g/g)	TFC co-extractant effectiveness ×10^−4^ (g/g)
1	–	50	VCO	2.0 ± 0.03^D^	-	0.6 ± 0.03^K^	1.1^K^	–	1.2^H^
2	–	50	CO	2.0 ± 0.02^D^	-	0.3 ± 0.02^M^	0.6^M^	–	0.6^I^
3	–	50	G	1.1 ± 0.02^F^	-	0.1 ± 0.04^N^	0.1^N^	–	0.1^K^
4	150	50	scCO_2_	0.8 ± 0.03^H^	1.23^G^	3.0 ± 0.01^G^	6.4^G^	1.2^G^	–
5	150	50	scCO_2_–VCO	5.3 ± 0.03^C^	15.16^C^	11.7 ± 0.01^C^	25.2^C^	39.3^B^	36.8^B^
6	150	50	scCO_2_–CO	1.4 ± 0.01^E^	3.23^D^	6.4 ± 0.05^D^	13.7^D^	5.5^E^	5.2^E^
7	150	50	scCO_2_–G	1.0 ± 0.04^G^	2.56^E^	3.1 ± 0.04^F^	6.6^F^	8.5^D^	8.0^D^
8	250	50	scCO_2_–VCO	5.3 ± 0.01^B^	15.73^B^	5.20 ± 0.04^E^	11.2^E^	16.7^C^	15.7^C^
9	350	50	scCO_2_–VCO	0.59 ± 0.04^J^	1.40^F^	2.11 ± 0.03^I^	4.5^I^	5.5^E^	3.1^F^
10	150	60	scCO_2_–VCO	0.35 ± 0.02^J^	1.16^H^	1.34 ± 0.03^J^	2.9^J^	0.7^H^	0.4^J^
11	250	60	scCO_2_–VCO	0.4 ± 0.03^J^	1.16^H^	2.43 ± 0.01^H^	5.2^H^	4.3^F^	2.4^G^
12	350	60	scCO_2_–VCO	0.11 ± 0.05^K^	0.89^I^	0.45 ± 0.04^L^	1.0^L^	0.2^I^	0.094^L^
13	–	50	MeOH	48.7 ± 0.02^A^	-	46.5 ± 0.0^A^	100^A^	–	–
14	150	50	scCO_2_-MeOH	3.4± 0.04^B^	24.70^A^	21.41± 0.02 ^B^	46.0^B^	192.23^A^	180.2^A^

20% mass ratio of MeOH or edible oil was mixed with the propolis. CO: corn oil; VCO: virgin coconut oil; G: ghee; scCO_2_: supercritical carbon dioxide; values that do not share the same superscript letter are significantly different (Tukey test, p-value < 0.05);—not available

### Extraction with supercritical carbon dioxide

**Fig 2:**
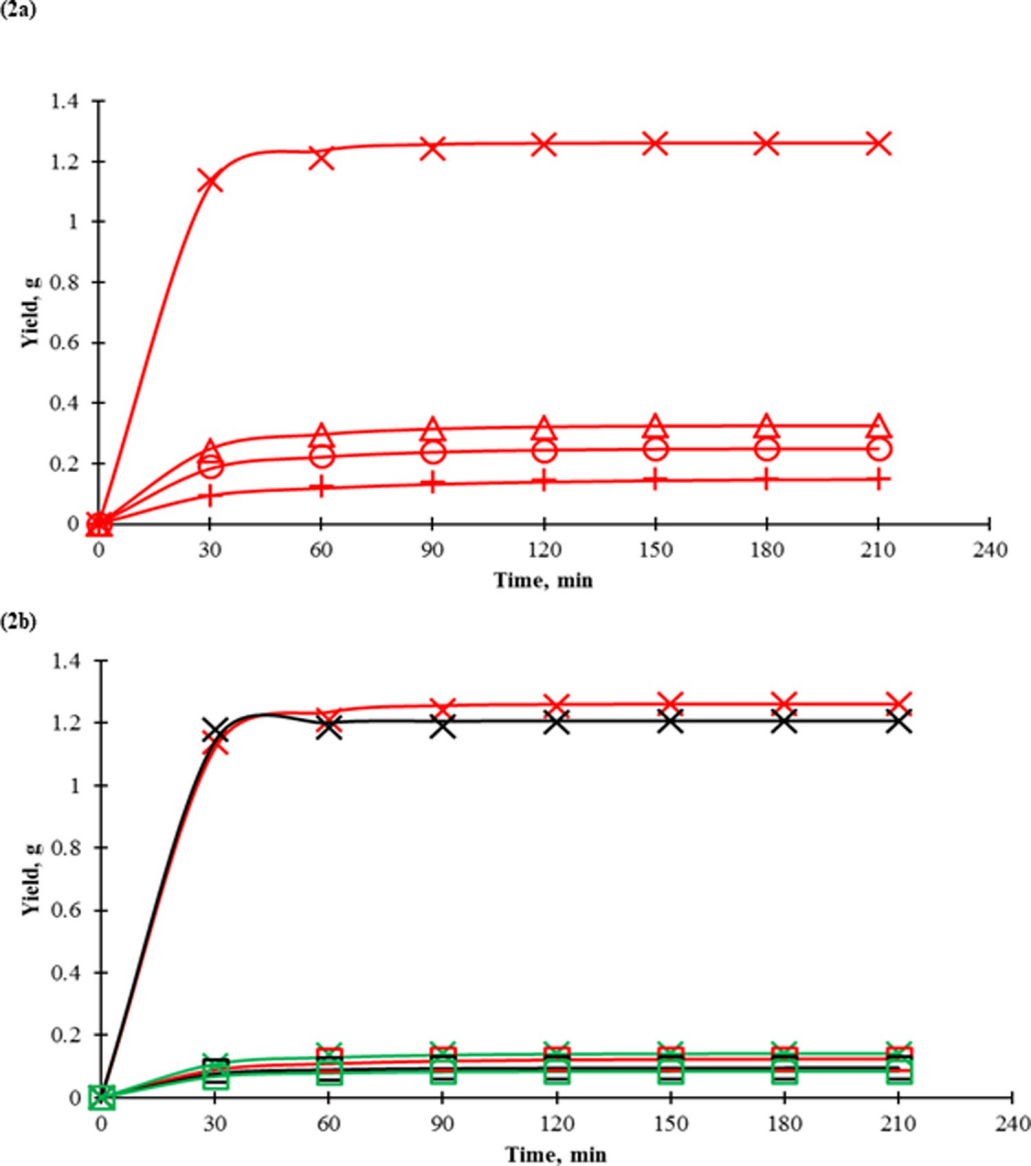
Extraction yield (co-extractant) were mixed with propolis used with supercritical carbon dioxide (scCO_2_ 25 g/min of carbon dioxide) (a). Solids line: Diffusion model, markers: experimental values, Red x scCO_2_–VCO,150 bar, 50°C; Red Δ scCO_2_–CO, 150 bar, 50°C; Red Ο scCO_2_–G, 150 bar, 50°C; Red ✦ scCO_2_-propolis, 150 bar, 50°C; Black × scCO_2_–VCO, 250 bar, 50°C; Green Χ scCO_2_–VCO, 350 bar, 50°C Red □ scCO_2_–VCO, 150 bar, 60°C; Black □ scCO_2_–VCO, 250 bar, 60°C; Green □ scCO_2_–VCO, 350 bar, 60°C.

As shown in Table 2, the yield obtained with scCO_2_ (Run 4) was low, which collected at early 30 min (Fig 2A), but concentration of TFC in extract was markedly increased as compared to the oil-based extract (Run 1, Run 2 and Run 3) and reported 6.4% of TFC recovery. The results showed that scCO_2_ was more selective than edible oils in extracting TFC from propolis, however, the TFC extraction effectiveness in the total mass of solvent, scCO_2_ (1.2 x10^-7^ g/g) was lesser than VCO (1.2 x 10^−4^ g/g). We noticed the residue (propolis) clumped in the extractor after extraction, possibly due to the high wax content (up to 30%, Table 1) in the propolis that tended to agglomerate when heated and pressurised, which caused the contact of the solute and solvent to become poor. This agglomeration might start to retard the extraction progress after 30 min of extraction (Fig 2A), which the obtained yield had reduced after that. Besides, the melting of beeswax might also be depressed to 56°C at 150 bar in scCO_2_ environment [37] which intensified the agglomeration. The study performed by Machado *et al*. [12], however manage to obtain 20.2 mg/g TFC in extract at higher pressure (250 bar) than this study.

### Extraction with edible oils and supercritical carbon dioxide

#### Effects of co-extractant

The extraction yields were noticeably increased when oils (co-extractant) were mixed with propolis (Run 5, Run 6 and Run 7) and extraction kinetics were shown in (Fig 2A). With oils, the first 80% of the extract was easier and faster obtained in the earliest stage (30 min) compared to without oil (Run 4), however, the last fraction was very slowly extracted in the later stage. The added edible oils could contribute to the first fraction of the extract which was obtained from the earlier stage. The slow extraction in the last stage might be attributed to the exhaustion of edible oils, the solutes retained in the matrix and wax in the propolis. The selectivity to TFC also increased markedly when co-extractant was used in scCO_2_ extraction, which showed an increase in TFC concentration in extract. Besides, the solvent and co-extractant effectiveness were improved up to 40 times compared to when sole scCO_2_ or oil was used. The report from Lee *et al*. also shows that oil could be used as co-extractant to enhance bioactive compounds recovery [38]. However, the effectiveness from this work still has a room of improvement as compared to when methanol was used as co-solvent (Run 14). Among the oils, VCO showed the most effective co-extractant as compared to G and CO in same extraction conditions. At the considered temperature (50°C), the viscosity of VCO (15.7 cp) is lower than that of CO (28 cp) and G (43 cp) [39], and the viscosity of oils are becoming lower in scCO_2_ [40], which helped in the extraction. As reported by Ghazani *et al*. [41], the high viscosities of edible oils have been regarded as a major reason for the low diffusion of solute into the oil phase. VCO has been categorised as medium triglyceride, which the major fatty acids are C10-C12, and has reported to be more polar and hydrophilic than oils that contain long chain fatty acids [38]. This made VCO was more suitable to extract polar compound such as flavonoid compounds.

#### Effect of pressure and temperature

As VCO was the best among the edible oils that functioned as co-extractant, it was chosen to study the effect of pressure and temperature on the extraction of TFC from propolis (Table 2). At the considered temperature, pressure had a negative effect on all the responses. At 50°C, increment of 150 bar to 250 bar, even though the extraction kinetics (Fig 2B) were almost the same, but the TFC selectivity had reduced to half. Besides, TFC recovery, solvent and co-extraction efficiencies were also clearly reduced. The extraction had become worse at 350 bar, concentration of TFC in extract, recovery, solvent effectiveness and co-extractant effectiveness were lower relative to 150 bar and 250 bar. The extract solubility decreased as pressure increased from 150 bar (15.7 × 10^−4^ (g/g)) to 350 bar (1.4 × 10^−4^ (g/g)). This trend was not observed in the other scCO_2_ extraction studies, as the pressure normally is direct proportional to the solubility of a solute [42]. In this study, the reduction might be attributed to propolis becoming soft at a high temperature [43] and becoming compressed on the application of high pressure, especially in scCO_2_ conditions as we discussed above. This compactness reduced the contact area of propolis, which in turn affected the mass transfer of the solute into the solvents [44]. The impact was more obvious when the temperature was increased to 60°C. In Run 10 (60°C), even though the pressure was only 150 bar, the yield and TFC in extract were much lower than at 50°C. The extraction had become worse as pressure increased to 350 bar. As shown in Fig 2B, the extraction curves were meager and almost overlaid each other, indicating a substantial intraparticle resistance [45] and illustrated the compactness of the propolis at a high temperature.

Even though the recovery of TFC with scCO_2_–VCO was only 25%, VCO is still a good option for use as a co-extractant, particularly in the food industry, because it avoids the residual organic solvents. The extract can be used directly as a food ingredient or food product.

#### Diffusion model

The OECs were correlated with diffusion model with the AARD of 1.6% (Fig 2); the matrix diffusivities are tabulated in Table 3. The fitting was performed with 72 data points. The matrix diffusivity of the solutes in the propolis without a co-extractant was the lowest (4.7 × 10^−11^ m^2^/s). As shown in Table 1, the propolis contained about 32% waxy materials and these waxy materials had limited solubility in scCO_2_ [46], which might slow down the diffusion of the solutes in the matrix. This implies that the intraparticle resistance in propolis was high. However, the solute matrix diffusivities in the propolis were improved when the co-extractants were used, and it was believed that the diffusion of the oil was the main effect. It had been reported that vegetable oil can dissolve the waxy material [46], therefore, the addition of the co-extractant to propolis and scCO_2_ might soften the waxy material, simultaneously recovering the solutes from propolis. Among the oils used, the diffusivity was the highest in scCO_2_-VCO which relate to viscosity of oil used in the study. As reported by [47], the diffusivity is inverse proportional to viscosity and as discussed earlier that VCO had the lowest viscosity as compared with CO and G. In particular, the use of scCO_2_–VCO at 150 bar to 250 bar at 50°C the diffusivity was increased and high yield was obtained because the matrix diffusivity was directly correlated to the mass transfer process [46]. Pressure has also found to be direct proportion to diffusivity by Lv *et al*. [47], however, in this study the diffusivity reduced markedly when pressure had increased from 150 bar to 350 bar at 50°C and low at 60°C in the studied pressure range. As discussed previously, the reduction in diffusivity in scCO_2_–VCO might relate to the compactness of the propolis due to the scCO_2_ and temperature.

**Table 3:** Matrix diffusivities of the solutes in the propolis determined by the diffusion model.

P (bar)	T (°C)	Solvents	*D* × 10^−11^, *m*^*2*^*/s*	AARD, %
150	50	scCO_2_	4.70	1.58
150	50	scCO_2_–VCO	14.90	
150	50	scCO_2_–CO	8.39	
150	50	scCO_2_–G	7.36	
250	50	scCO_2_–VCO	21.35	
350	50	scCO_2_–VCO	8.37	
150	60	scCO_2_–VCO	6.87	
250	60	scCO_2_–VCO	9.85	
350	60	scCO_2_–VCO	9.85	

20% mass ratio edible oil to propolis. VCO: virgin coconut oil; CO: corn oil; G: ghee; scCO_2_: supercritical carbon dioxide; supercritical carbon dioxide: 150 bar and 50°C

### Identification of flavonoids in the extract

Table 4 shows the tentative identified flavonoid compounds in the propolis extract. The combination of scCO_2_–VCO managed to extract more flavonoids than scCO_2_ and VCO. In all, 13 flavonoids were tentatively identified in the extract from scCO_2_–VCO and 12 flavonoids from methanol. These results showed that VCO could be used as co-extractant in extraction flavonoids from propolis. These flavonoids consisted of flavonol, isoflavonoid, flavones, flavanones, O-methylated isoflavone, isoflavones, and homoisoflavanone.

**Table 4:** LC-MS analysis of the flavonoid compounds extracted from propolis via various extraction methods.

Tentative Flavonoid	Retention time of the peaks in the chromatograms (min)				Molecular weight (g/mol)	Group of flavonoids
	Methanol	scCO_2_-VCO	VCO	scCO_2_		
Izalpinin	6.92	6.67	-	-	284.07	Flavonol
Genistein	7.58	7.07	-	-	270.05	Isoflavonoid
Chrysin	7.89	7.64	-	8.01	254.06	Flavones
5,7-Dihydroxy-4’-methoxy-8-phenylflavanone	-	8.36	-	-	300.30	Flavanones
Kaempferol	8.91	9.54	-	-	286.23	Flavonols
Fisetin	10.66	10.11	-	-	286.05	Flavonols
Irigenin	9.79	-	-	-	360.08	O-methylated isoflavone
Ichthynone	10.21	8.90	-	-	408.12	Isoflavones
Brazilien	10.88	13.37	-	10.63	-	-
Koparin	10.64	-	-	-	300.26	Isoflavonoid
5,7-dimethoxyisoflavone	11.60	10.79	-	11.52	282.29	Isoflavones
Sappanone A 7-methyl ether	13.02	11.42	-	-	298.29	Flavanone
Isotectorigenin	13.40	12.29	-	-	328.30	Homoisoflavanone

VCO-virgin coconut oil; 20% (w/w) of MeOH or VCO was mixed with propolis powder at 50°C for 2.5 h; Extraction conditions: supercritical carbon dioxide (scCO_2_) 150 bar, 50°C;—compound not detected.

The bioactivities of these compounds such as genistein demonstrate inhibition of prostate cancer cells [48], chrysin in oral cavity diseases [49], sappanone A as a therapeutic agent for asthma [50], and fisetin in suppressing NF-κB activation in bladder cancer T24 cells and prostate cancer PC3 cells [51]. As compared to the recent report from Fachri *et al*. [52] the flavonoids compounds (myricetin, pinobanksin quercetin, kaempferol, galangin and chrysin) are different with this study. It was clearly recognized about the propolis differentiations related to their regions, flora and production conditions [53]. The chromatograms of each method are provided in the S1 Appendix.

## Conclusion

In this study, edible oils, namely virgin coconut oil (VCO), corn oil (CO), and ghee (G), were used as the co-extractant for assisting supercritical carbon dioxide (scCO_2_) in extracting flavonoids from propolis. Overall, the addition of edible oils improved the extraction process; in particular, scCO_2_–VCO showed the highest total flavonoid content (TFC), recovery, and efficiency. However, when high pressures (250 bar and 350 bar) and temperature (60°C) were used, the performance deteriorated markedly. According to the overall extraction curves, the rapid extraction in the early stage might be attributed to the edible oil and the slow extraction in the later stage due to the exhaustion of the edible oil. The diffusion model correlated the OECs with AARD of 1.6%, and the matrix diffusivities of the solute in the presence of a co-extractant were estimated to be 6.9 × 10^−11^ m^2^/s to 21.35 × 10^−11^ m^2^/s. The tentative flavonoids found in the scCO_2_–VCO extract were mainly flavones, flavonols, flavanones, and isoflavonoids.

## Supporting information

S1 Appendix (DOCX)
