# Supplementary material for: Edible oils as a co-extractant for the supercritical carbon dioxide extraction of flavonoids from propolis
Source: PLoS One. 2022 Apr 12;17(4):e0266673. doi: 10.1371/journal.pone.0266673 (PMC9004773; doi:10.1371/journal.pone.0266673)
Supplement: S1 Appendix — (DOCX) [file pone.0266673.s001.docx]

**Edible oils as a co-extractant for the supercritical carbon dioxide extraction of flavonoids from propolis**

Parveen Devi Pattiram^1^, Faridah Abbas^1^, Norhidayah Suleiman^1^, Ezzat Mohamad Azman^1^, Gun Hean Chong^1,2^*

^1^ Faculty of Food Science and Technology, Universiti Putra Malaysia, 43400 UPM Serdang, Selangor Malaysia

^2^ Supercritical Fluid Center, Faculty of Food Science and Technology, Universiti Putra Malaysia, 43400 UPM Serdang, Selangor Malaysia

* Corresponding author

Email: [gunhean@upm.edu.my](mailto:gunhean@upm.edu.my)

**Appendix 1: LCMS chromatogram of tentative flavonoids compound in VCO, propolis used with supercritical carbon dioxide (scCO_2_ 25 g/min of carbon dioxide) at 150 bar, 50 ^°^C.**


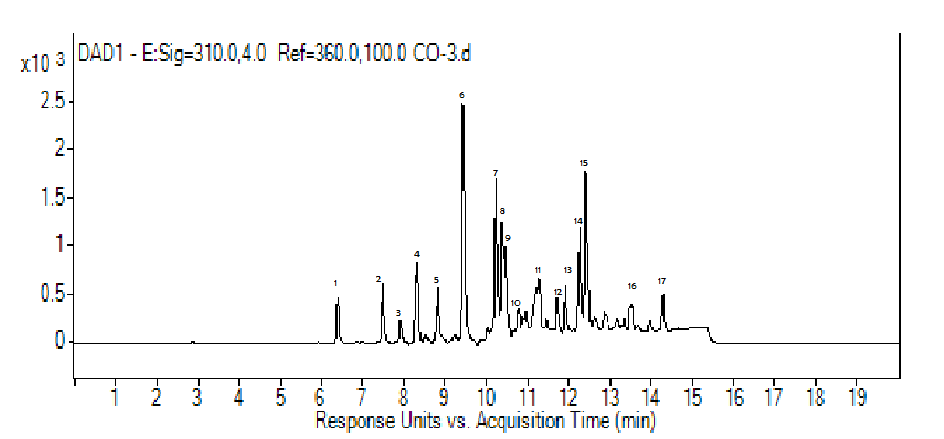


| Peak No. | Tentative compound | Retention Time (min) | Molecular weight (g/mol) |
| --- | --- | --- | --- |
| 1 | Izalpinin | 6.692 | 284.07 |
| 2 | Genistein | 7.076 | 270.05 |
| 3 | Chrysin | 7.642 | 254.06 |
| 4 | 5,7-Dihydroxy-4'-methoxy-8-phenylflavanone | 8.366 | 300.30 |
| 6 | Ichthynone | 8.909 | 408.12 |
| 7 | Irigenin | 9.514 | 360.08 |
| 8 | Kaempferol | 9.542 | 286.23 |
| 9 | Fisetin | 10.114 | 286.05 |
| 10 | Koparin | 10.637 | 300.26 |
| 11 | 5,7-dimethoxyisoflavone | 10.791 | 282.29 |
| 12 | Sappanone A 7-methyl ether | 11.421 | 298.29 |
| 13 | Isotectorigenin | 12.294 | 328.30 |
| 16 | Brazilein | 13.373 | - |

**Appendix 2: LCMS chromatogram of tentative flavonoids compound in propolis used with supercritical carbon dioxide (scCO_2_ 25 g/min of carbon dioxide) at 150 bar, 50 ^°^C.**


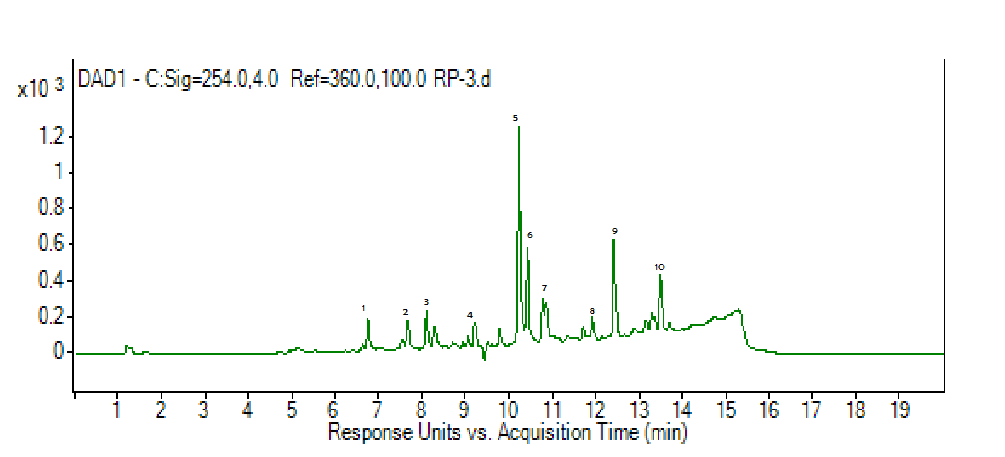


| Peak No. | Tentative compound | Retention Time (min) | Molecular weight (g/mol) |
| --- | --- | --- | --- |
| 2 | Chrysin | 8.013 | 254.06 |
| 5 | Ichthynone | 10.219 | 408.12 |
| 7 | Koparin | 10.634 | 300.26 |
| 8 | 5,7-dimethoxyisoflavone | 11.529 | 282.29 |

**Appendix 3: LCMS chromatogram of tentative flavonoids compound in solvent extraction (MeOH) propolis at 50 ^°^C.**


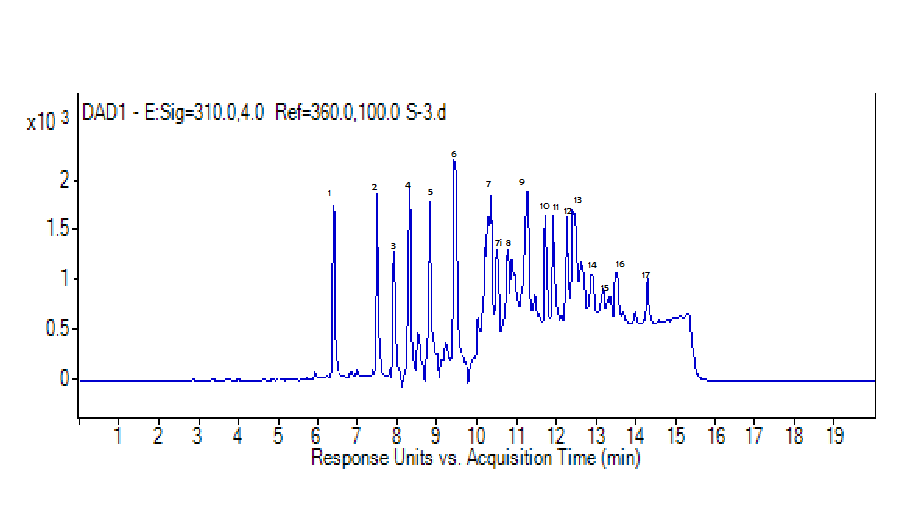


| Peak No. | Tentative compound | Retention Time (min) | Molecular weight (g/mol) |
| --- | --- | --- | --- |
| 1 | Izalpinin | 6.923 | 284.07 |
| 2 | Genistein | 7.586 | 270.05 |
| 3 | Chrysin | 7.891 | 254.06 |
| 5 | Kaempferol | 8.918 | 286.23 |
| 6 | Irigenin | 9.797 | 360.08 |
| 7 | Koparin | 10.649 | 300.26 |
| 8 | Fisetin | 10.661 | 286.05 |
| 9 | Brazilein | 10.882 | - |
| 10 | Ichthynone | 11.143 | 408.12 |
| 12 | 5,7-dimethoxyisoflavone | 11.601 | 282.29 |
| 14 | Sappanone A 7-methyl ether | 13.021 | 298.29 |
| 15 | Isotectorigenin | 13.402 | 328.30 |

**Appendix 4: LCMS chromatogram of tentative compound in VCO, propolis extraction at 50 ^°^C.**


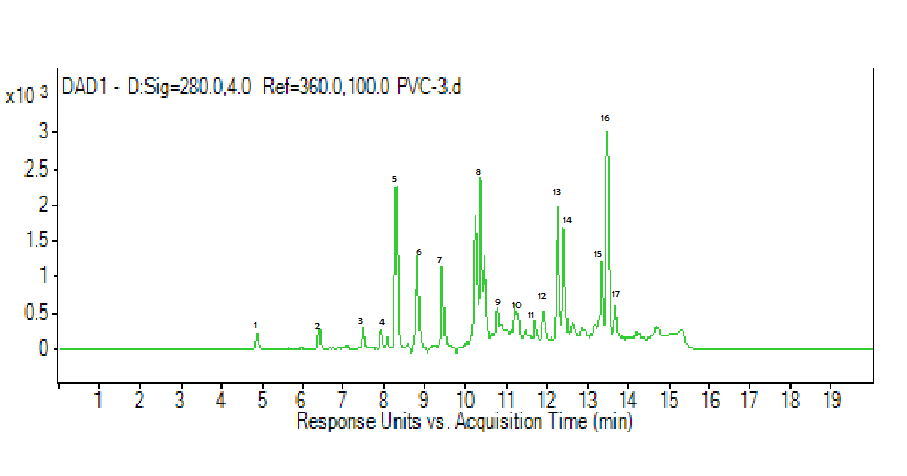


**Appendix 5: LCMS chromatogram of tentative flavonoids compound in VCO (Control).**


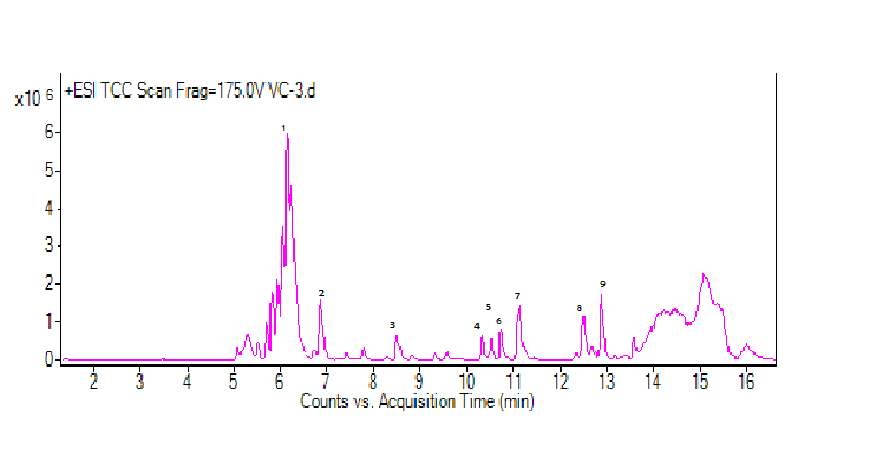


Tentative flavonoids compounds were not detected from LCMS chromatogram by using the internal library, Knapsack core system library and National Institute of Standard and Technology (NIST) in VCO, propolis and VCO (control).
